# Cutaneous Adverse Drug Reactions Associated with SGLT2 Inhibitors

**DOI:** 10.3390/jcm14010188

**Published:** 2024-12-31

**Authors:** Alexandra Laura Mederle, Patrick Dumitrescu, Claudia Borza, Nilima Rajpal Kundnani

**Affiliations:** 1 Faculty of Medicine, “Victor Babeș” University of Medicine and Pharmacy, 300041 Timisoara, Romania; 2 Discipline of Pathophysiology, Department of Functional Science, “Victor Babeș” University of Medicine and Pharmacy, 300041 Timisoara, Romania; 3 Center for Translational Research and Systems Medicine, “Victor Babeș” University of Medicine and Pharmacy, 300041 Timisoara, Romania; 4 Centre of Cognitive Research in Pathological Neuro-Psychiatry NEUROPSY-COG, “Victor Babeș” University of Medicine and Pharmacy, 300041 Timisoara, Romania; 5 Department of Cardiology—Internal Medicine and Ambulatory Care, Prevention and Cardiovascular Recovery, “Victor Babeș” University of Medicine and Pharmacy, 300041 Timisoara, Romania; knilima@umft.ro; 6 Research Centre of Timișoara Institute of Cardiovascular Diseases, “Victor Babeș” University of Medicine and Pharmacy, 300041 Timisoara, Romania

**Keywords:** SGLT2 inhibitors, cutaneous adverse drug reactions, diabetes mellitus, antidiabetics, oral hypoglycemic agents, diabetes mellitus

## Abstract

Diabetes is a complex global healthcare burden involving multiple organ systems with its prevalence on the rise. SGLT2 inhibitors enhance glucose excretion. The objective of our literature review was to determine the association between cutaneous adverse drug reactions (CADRs) and the use of SGLT2 inhibitors. We collected data on CADRs related to the use of SGLT2 inhibitors from all available published articles and studied their details to understand the patterns of their association. PubMed, Cochrane, Google, and Embase were searched for relevant articles. A total of 37 papers were included and studied. Most articles were case reports followed by pharmacovigilance studies, case series, and reviews. The cutaneous findings ranged from benign eruptions to severe reactions. The available literature suggests a strong link between the use of SGLT2 inhibitors and Fournier’s gangrene/necrotizing fasciitis. T2DM patients using SGLT2 inhibitors have also developed fixed drug eruptions, drug-induced pruritus, and Sweet syndrome/acute febrile neutrophilic dermatosis, among other skin lesions. We found that SGLT2 inhibitors present a risk of developing CADRs. Raising awareness among healthcare providers regarding CADRs to SGLT2 inhibitors can reduce complications, minimize hospitalizations, and improve patient care in the vulnerable population of diabetes patients.

## 1. Introduction

Type 2 diabetes mellitus (T2DM) is a global health issue with an estimated global prevalence of 463 million people, with its incidence on the rise along with multiple clinico-pathological repercussions during its treatment and complications [1]. Sodium–glucose cotransporter 2 (SGLT2) inhibitors are a relatively newer emerging group of oral hypoglycemic agents in use for the treatment of T2DM [2] as selective and reversible inhibitors of the SGLT2 co-transporter [3]. On the proximal convoluted tubule of the kidneys, SGLT1 and SGLT2 co-transporter proteins reabsorb sodium and glucose from the glomerular filtrate, and, in T2DM, the SGLT2 are upregulated, thereby inducing further hyperglycemia that is independent of insulin. SGLT2 inhibitors act on them to enhance the excretion of sugar and salt from the body via urine, resulting in glycosuria. This group works in an insulin-independent manner to lower glycemia and, hence, the risk of hypoglycemia is marginal. There is also some evidence of improvement of beta cell function by SGLT2 inhibitors, which further helps in lowering HbA1c [4]. SGLT2 inhibitors include canagliflozin, dapagliflozin, empagliflozin, ertugliflozin, bexaglifloxin, ipragliflozin, and sotagliflozin [5].

This group is the preferred group for prescribing to type 2 diabetes mellitus (T2DM) patients suffering from heart failure and chronic kidney disease [6,7]. SGLT2 inhibitors have been approved by the United States Food and Drug Administration for the management of type 2 diabetes mellitus over at least the past 10 years [2]. Canagliflozin, dapagliflozin, empagliflozin, and ertugliflozin are the SGLT2 inhibitors to have received United States Food and Drug Administration approval in chronological order.

Studies have shown that SGLT2 inhibitors improve life expectancy and reduce the risk of worsening coronary artery disease, heart failure, and chronic kidney disease [8,9]. SGLT2 inhibitors are effective in glycemic control, the reduction in the risk for major adverse cardiovascular events [8], and the reduction in kidney disease progression [9] in T2DM. Importantly, SGLT2 inhibitors do not lead to drug-induced hypoglycemia in T2DM patients, and have also shown adjunctive effects in the management of hypertension, diabetic nephropathy, and lower risks of arrhythmias, and improved the bioavailability of endothelium-derived nitric oxide [10]. The benefits of SGLT2 inhibitors include the prevention of microvascular and macrovascular complications arising from uncontrolled diabetes, a decrease in microalbuminuria, other nephroprotective effects, weight loss, and improved outcomes in heart failure [11,12].

This group of drugs was launched with claims of being relatively free from major side effects. Known adverse effects of SGLT2 inhibitors are diabetic ketoacidosis, urinary tract infections, genital infections, cutaneous adverse drug reactions (CADRs), bone fractures, and rare reports of cancer [13,14,15,16,17,18]. There are reports of euglycemic diabetic ketoacidosis in T2DM patients using SGLT2 inhibitors [19,20,21,22], with approximately 0.1% overall incidence [23], especially in patients with lower body mass index and decreased glycogen stores [24]. SGLT2 receptors are located in proximal renal tubules and they reabsorb sodium and glucose [25]. If this process is blocked, the excretion of sodium and glucose into the urine will be enhanced and will work against hyperglycemia [25]. But, this leads to high glucose levels in urine, which leads to the risk of urinary tract infections and genital fungal infections [13,25].

Diabetes mellitus (DM), as a disease, has a known association with cutaneous pathological processes with varying severity, offering insight into the patients’ glycemic control [26], with 79.2% of patients facing some skin issue or other, commonly ranging from cutaneous infections and xerosis to inflammatory skin diseases [27]. These skin issues are nail discoloration, acrochordons, acanthosis nigricans, skin tags, diabetic dermopathy, rubeosis faciei, pruritus, granuloma annulare, necrobiosis lipoidica, scleroedema diabeticorum, bullosis diabeticorum, perforating dermatosis, calciphylaxis, eruptive xanthoma, and infections [28], as well as local reactions, contact dermatitis, and lipohypertrophy at insulin injection sites. Hyperglycemia, insulin deficiency, insulin resistance, and non-enzymatic glycation are some important factors that lead to biochemical alterations in the form of microangiopathy, neuropathy, impaired wound healing, and immune response changes [29], as well as the suppression of proliferation and migration of keratinocytes and fibroblasts [30], to contribute to the cutaneous complications. To make things complicated, diabetes patients require one or multiple oral hypoglycemic agents on a daily basis for several years, and all the oral hypoglycemic agents have been linked with one CADR or other [14,31]. CADRs are undesirable skin manifestations arising from systemic drug administration and are also known as toxidermia. CADRs are classified as immunologic and non-immunologic based on their underlying mechanism [32]. The immunologic CADRs consist of either immediate or delayed immunologic mechanisms mediated by cellular or humoral immune responses. As a public health concern, they affect about 1% to 3% of multi-medicated patients [33]. CADRs are managed using topical corticosteroids, oral antihistaminics, and the discontinuation of the involved drug. CADRs are recognized as major global health concerns due to the financial burden on healthcare systems, with a potential source for drug non-compliance and having an important role in patients’ depression and psychological well-being [34,35,36].

## 2. Materials and Methods

**Figure 1:**
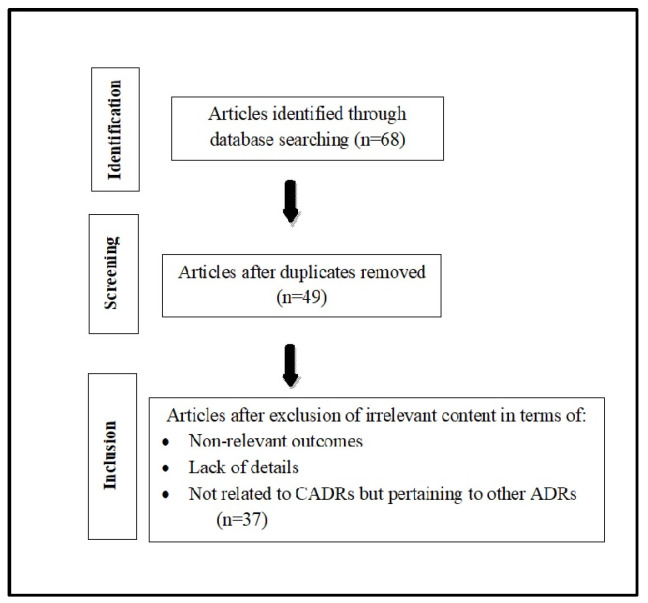
Flowchart of identification, screening, and inclusion of articles.

PubMed, Cochrane, Google, and Embase were used to search for articles that discuss CADR to the SGLT2 inhibitor group of diabetes medications. Key words and titles searched included “diabetes medication”, “antidiabetic drugs”, “oral hypoglycemic agents”, “adverse drug reactions”, “cutaneous”, “skin”, “reactions”, “allergic reactions”, “toxidermia”, “diabetes”, “sodium-glucose cotransporter 2 inhibitors”, “SGLT2 inhibitors”, “canagliflozin”, “dapagliflozin”, “empagliflozin”, “ipraglflozin”, “bexagliflozin”, and “sotagliflozin”. The flowchart of the process of identification, screening, and inclusion of articles is summarized in Figure 1. Key features of various SGLT2 inhibitor drugs are summarized in Table 1.

## 3. Results and Discussion

### 3.1. Overview

A total of 37 papers were included and studied. We found articles highlighting skin-related adverse events after using SGLT2 inhibitors, and they were studied in detail and summarized in Table 2. It was found that SGLT2 inhibitors present a major risk of developing a CADR. A strong link was observed between the use of SGLT2 inhibitors and Fournier’s gangrene/necrotizing fasciitis. T2DM patients using SGLT2 inhibitors also developed fixed drug eruptions, drug-induced pruritus, and Sweet syndrome/acute febrile neutrophilic dermatosis, among other skin lesions. These are further elaborated in the discussion section. The least number of cases had some CADR in association with ertugliflozin and sotagliflozin, but this may be because the drug is relatively new, with a lower number of patients exposed to it and a shorter duration of use by the patients.

### 3.2. Fournier’s Gangrene/Necrotizing Fasciitis: A Growing Pool of Cases

Chi and Lim-Tio [37], Kumar et al. [38], Omer et al. [39], Onder et al. [40], Rodler et al. [42], Fadini et al. [16], Bersoff-Matcha et al. [66], Nagano et al. [66], Elshimy et al. [43], Ellegard and Prytz [45], Elbeddini et al. [44], Hu et al. [50], Wang et al. [51], Garcia-Garcia et al. [46], Kasbawala et al. [47], Moon et al. [48], Tran et al. [52], and Suciu et al. [49] reported a large number of Fournier’s gangrene cases in patients taking SGLT2 inhibitors.

Chi and Lim-Tio [37] reported one of the earliest case reports of a 67-year-old male with Fournier’s gangrene developed 3 weeks after the initiation of dapagliflozin. Fourteen months after commencing 25 mg/day empagliflozin, a 67-year-old male with a 10-year history of T2DM presented in an emergency with genital pain, swelling, and necrosis, and was diagnosed with Fournier’s gangrene [38]. He underwent emergency surgical debridement and antibiotic treatment. A pharmacovigilance study conducted by Fadini et al. [16] studied the association between SGLT2 inhibitors and the development of Fournier’s gangrene and other severe genital cutaneous adverse events. Spontaneous reports of adverse events submitted to the FDA Adverse Event Reporting System (FAERS) database and the European Medicines Agency’s EudraVigilance database were studied to identify 123 cases of Fournier’s gangrene and 457 cases of other severe genital cutaneous adverse events associated with SGLT2 inhibitors, including canagliflozin, dapagliflozin, and empagliflozin, with predominantly male patients (90%) with a median age of 57 years [16].

According to Bersoff-Match et al. [66], 55 cases of Fournier’s gangrene were reported up until January 2019, which is a large volume of cases. Bersoff-Matcha [66] analyzed case reports from the FAERS database between 2013 and 2018 and identified 55 cases of Fournier’s gangrene associated with SGLT2 inhibitors, including canagliflozin, dapagliflozin, and empagliflozin. With 94% of the reports in males and a median age of 57 years, the median time from the commencement of the SGLT2 inhibitor to the onset of symptoms was 30 days, and canagliflozin was, again, the most commonly accounted for drug with 63% of cases [66]. Nagano Y. et al. [41] reported a 34-year-old male with T2DM developing Fournier’s gangrene after being placed on treatment with a regular dose of 10 mg/day of empagliflozin for about 5 months. Empagliflozin was discontinued, and the patient underwent surgical debridement and antibiotic treatment.

Elshimy et al. [43] reported Fournier’s gangrene with severe genital pain, swelling, and necrosis in a 57-year-old male with T2DM 10 days after commencing canagliflozin. He underwent multiple surgeries and hospitalizations before recovering. Elbeddini et al. [44] reported Fournier’s gangrene in a 72-year-old male with T2DM while on canagliflozin. He also experienced substantial morbidity. Ellegård et al. [45] reported Fournier’s gangrene in a 52-year-old female with T2DM taking dapagliflozin for 1.5 years. She was on prednisolone after a past adrenalectomy, and uncontrolled T2DM may also have contributed [45].

Hu et al. [50] conducted a pharmacovigilance study aimed at investigating the relationship between SGLT2 inhibitors including canagliflozin, dapagliflozin, and empagliflozin and Fournier’s gangrene using data from the FAERS database from 2013 to 2019. They identified 71 cases of Fournier’s gangrene with a reporting rate of 0.14 per 100,000 patient-years. With 83% of the reports in males and median age of 57 years, canagliflozin alone accounted for 46% of the cases [50].

### 3.3. Fournier’s Gangrene/Necrotizing Fasciitis: Nested Case-Control Study and Review of Post-Marketing Case Reports

Wang et al. [51] conducted a nested case-control study to investigate the link between SGLT2 inhibitors and the risk of hospitalization for Fournier’s gangrene. Data from a large US-based insurance claims database was analyzed to identify 14,581 patients hospitalized for Fournier’s gangrene between 2013 and 2018, and these were matched with 145,810 non-hospitalized controls. With an odds ratio (OR) of 3.64 (95% CI, 2.63–5.04), SGLT2 inhibitors were found to be associated with a higher risk of hospitalization for Fournier’s gangrene [51]. An important finding was that the risk of hospitalization was highest in the first 30 days of using SGLT2 inhibitor with OR 5.65 (95% CI, 3.23–9.89) [51].

Tran et al. [52] provided a review of case reports and post-marketing cases identified from FAERS database, and concluded a strong link between SGLT2 inhibitors and Fournier’s gangrene. Tran et al. [52] conducted a comprehensive review of case reports and spontaneous post-marketing cases of Fournier’s gangrene associated with SGLT2 inhibitors as reported to the FAERS database. As a result, 55 cases of Fournier’s gangrene were identified as associated with SGLT2 inhibitors in patients having T2DM, including canagliflozin, dapagliflozin, and empagliflozin, with the vast majority of cases reported in males (94%) with a median age of 57 years, and the median time to onset of symptoms as 30 days after initiating SGLT2 inhibitor therapy. The authors infer that there is a need for a stricter follow-up of any patient commencing SGLT2 inhibitor for at least the first 30 days.

### 3.4. Fournier’s Gangrene/Necrotizing Fasciitis Case Reported in Romania

Suciu et al. [49] presented probably the first case of Fournier’s gangrene from Romania in a patient on SGLT2 inhibitor. They reported Fournier’s gangrene in a 65-year-old male with T2DM having severe genital pain and swollen testicles after being on an unspecified SGLT2 inhibitor for 6 months prior to the presentation. The patient later on presented with congestive heart failure and severe left ventricular dysfunction with 10–15% left ventricular ejection fraction and underwent coronary angioplasty; however, this may or may not be directly attributed to the commencement and discontinuation of the SGLT2 inhibitor.

### 3.5. Fournier’s Gangrene/Necrotizing Fasciitis—Underlying Mechanisms and Management

Fournier’s gangrene is a bacterial infection of the subcutaneous tissue of the perineum. Fournier’s gangrene is characterized by necrotizing fasciitis localized in the perineal region. It may present with foul-smelling discharge, abscess, crepitus of inflamed tissues, edema, redness, nausea, and pain [70]. It is often painful, but sometimes pain sensations are not adequately reported by patients with diabetic neuropathy. This necrotizing fasciitis of the perineum is rapidly progressive and often a urological emergency requiring urgent surgical debridement and broad-spectrum antibiotics [71]. It is known to rapidly progress into sepsis and has high mortality rates [72,73]. Fournier’s gangrene carries higher mortality rates in the presence of co-morbidities such as compromised immune system, diabetes, cardiovascular disease, and kidney disease [74]. Obesity, male gender, smoking, alcohol abuse, advancing age [75,76], immunosuppression, HIV infection, diabetes, and end-stage renal or liver disease [75], as well as perineal issues such as urine leak, a history of perineal surgery, or iatrogenic causes, increase the risk of development of Fournier’s gangrene [51,77]. It is more common in males. Fournier’s gangrene infection is polymicrobial in nature that can involve aerobic as well as anaerobic bacteria species. Commonly found species are methicillin resistant *Staphylococcus aureus* (MRSA), *Bacteroides* species, *Escherichia coli*, *Pseudomonas aeruginosa*, *Streptococcus* species, and *Prevotella* species [78]. SGLT2 inhibitors enhance glucosuria from the proximal convoluted tubule and prevent glucose reabsorption [3], which leads to elevated glucose levels in the urine and poses a risk of developing Fournier’s gangrene [13]. Although the exact mechanism is unclear, the increased urine glucose may be the culprit by creating a favorable environment for bacterial overgrowth. Early recognition, drug discontinuation, and treatment are crucial to prevent substantial morbidity and mortality.

Fournier’s gangrene typically requires prolonged treatment with broad-spectrum antibiotics and often surgical intervention [78,79]. It is treated with broad-spectrum antibiotics along with surgical debridement [72]. There is an interesting finding regarding sex distribution and the risk of developing Fournier’s gangrene in those patients that are on dapagliflozin. According to one study, 98% of Fournier’s gangrene patients are males [80,81]. On the contrary, another study found that 43.4% of Fournier’s gangrene patients are females [82]. This discrepancy suggests that there may be a possibility of the missed diagnosis or under-diagnosis of Fournier’s gangrene in females. In 2018, the FDA has issued a black box warning about multiple cases of Fournier’s gangrene in patients taking SGLT2 inhibitors [66]. It took any time between less than 2 weeks [43] to 6 years [44] to develop Fournier’s gangrene after using SGLT2 inhibitors. This suggests that there have to be several factors such as co-morbidities and risk factors at play in order to develop Fournier’s gangrene using SGLT2 inhibitors.

### 3.6. Fixed Drug Eruptions

In a study by Raschi et al. [65], international spontaneous reporting systems were examined including the WHO VigiBase (2009–2016), the FAERS (2013–2016), and the European Medicines Agency database (2012–2016). They analyzed 23,516 reports of adverse events related to SGLT2 inhibitors, with the most common adverse events being urinary tract infections, hypoglycemia, renal disorders, hypotension, and skin reactions. Among the skin reactions, fixed drug eruptions were among the most common findings.

Keskin et al. [53] reported a case of fixed drug eruptions after using dapagliflozin in addition to metformin and gliclazide for T2DM. A middle-aged female with T2DM presented with well-defined erythematous plaques on her trunk and limbs after commencing the intake of a 10 mg once-a-day dose of dapagliflozin. The lesions appeared two weeks after starting the dose and resolved upon discontinuation. A skin biopsy revealed epidermal necrosis and lymphocytic infiltration and patch testing confirmed dapagliflozin as the cause. Fixed drug eruptions are a cell-mediated delayed type of drug reaction that present as single or multiple round sharply demarcated erythematous edematous plaques anywhere on the body. This report highlighted SGLT2 inhibitors as a rare but potential cause of a fixed drug eruption, and there has to be awareness of this possibility. Early recognition and discontinuation of the offending drug are crucial in such a case.

There are more reports of fixed drug eruptions with SGLT2 inhibitors. Damiani et al. [54] reported a rare case of fixed drug eruptions associated with canagliflozin, where a 64-year-old female developed multiple well-defined erythematous plaques on her limbs and trunk 15 days after starting canagliflozin. A skin biopsy revealed epidermal necrosis and lymphocytic infiltration. Interestingly, during the episode, the patient’s glucose levels were under control. The lesions resolved upon discontinuation of the medication. Saito-Sasaki et al. [55] reported the first documented case of fixed drug eruptions by ipragliflozin. After starting ipragliflozin, a 67-year-old Japanese female developed multiple erythematous macules and papules on her trunk and extremities, with lymphocytic infiltration and epidermal spongiosis on the skin biopsy.

Yabe et al. [56] and Sakaeda. et al. [57] noted skin reactions with generalized rashes, eruptions, urticaria, erythema, and eczema after the use of ipragliflozin. Using 3D silico docking simulation, it was suggested that ipragliflozin is stored in the skin tissue, and interaction with melanin might be at play; hence, disturbances in skin homeostasis could have led to such lesions [57].

### 3.7. SGLT2-Inhibitor-Induced Pruritus

There are reports of pruritus with hyperpigmented maculopapular rashes in T2DM patients after starting SGLT2 inhibitors. Vasapollo et al. [58] reported a case of drug-induced pruritus in a 61-year-old female who developed intense severe pruritus after commencing canagliflozin. Clinical and laboratory findings excluded the presence of systemic or skin diseases. After discontinuing canagliflozin, there was a remission of pruritus. In another case report, Yau et al. [59] described a rare case of pruritic rash associated with empagliflozin in a 65-year-old female. There was a development of a pruritic rash on her trunk and extremities within 2 weeks of starting empagliflozin, which progressed to erythematous papules and plaques. A skin biopsy revealed lymphocytic infiltration and epidermal spongiosis. Ali et al. [60] reported a case of severe acute pruritus along with symmetrically distributed hyperpigmented scars and papules seen all over the body within 2–4 weeks of initiating the use of dapagliflozin in a 47-year-old female.

Most cases of drug-induced pruritus have been described in the literature as arising due to drug-induced cholestasis, secondary to pre-existing skin lesions, or from raised bradykinin levels [83]. However, in this case, there was no cholestasis or drug-induced liver injury and no pre-existing lesions. Angiotensin-ii reabsorbs sodium on the proximal convoluted tubule of the nephron by activating certain proteins and enzymes, the location where the SGLT2 co-transporter is also expressed. There may be a synergistic relationship between renin angiotensin aldosterone system (RAAS) blockade and SGLT2 inhibition and this may raise bradykinin levels and other proteins/enzymes [84,85]. A combination of angiotensin-converting enzyme inhibitors and SGLT2 inhibitors may tend to raise the angiotensin-converting enzyme 2:angiotensin-converting enzyme ratio, which, in turn, would lead to pruritus [83]. SGLT2 inhibitor use leads to increased glycosuria and also releases histamine and other mediators; however, the underlying mechanism is not clear. As seen in raised bradykinin, the incidence of dry cough in patients commencing SGLT2 inhibitors is not observed in daily medical practice; hence, this theory requires further research. Pruritus affects a noteworthy number of patients taking SGLT2 inhibitors, so it cannot be simply neglected [65]. Further studies should aim to understand the pathophysiology and explore the RAAS blockade theory and optimal management of SGLT2-inhibitor-induced pruritus.

### 3.8. Sweet Syndrome/Acute Febrile Neutrophilic Dermatosis

Mattis et al. [61] reported a rare case of acute febrile neutrophilic dermatosis, also known as Sweet syndrome, induced by dapagliflozin. Within 2 weeks of commencing dapagliflozin, a 67-year-old female with T2DM developed fever, arthralgia, and tender erythematous plaques on her limbs and face. A skin biopsy revealed neutrophilic infiltration and leukocytoclastic vasculitis, along with raised ESR and CRP. Upon discontinuing dapagliflozin, her symptoms subsided.

Acute febrile neutrophilic dermatosis is an overall rare condition and an even rarer drug-induced adverse event. It is characterized by the sudden onset of painful erythematous nodules, fever, arthralgia, and systemic symptoms such as headaches [86,87]. It is a non-infectious disorder characterized by neutrophilic infiltration of the epidermis, dermis, or hypodermis with or without true vasculitis [88]. Sweet syndrome is a non-vasculitic neutrophilic dermatosis similar to pyoderma gangrenosum, pustular psoriasis, keratoderma blennorrhagicum, rheumatoid neutrophilic dermatosis, Behcet disease, acne fulminans, familial Mediterranean fever, and syndromes of synovitis, acne, pustulosis, hyperostosis, and osteomyelitis (SAPHO) [88,89]. Although it is a clinical diagnosis, confirming this diagnosis relies on neutrophilic infiltrate in the histological examination of the lesions [90]. Immune-mediated reactions, cytokine imbalance, and neutrophil activation are among the potential underlying mechanisms behind Sweet syndrome. Sweet syndrome is reported in the literature as induced by a variety of drugs, and can co-exist with inflammatory and autoimmune diseases [91]. Any patient on SGLT2 inhibitors should be watched for the triad of fever, arthralgias, and skin lesions for the early detection of neutrophilic dermatosis.

### 3.9. Stevens–Johnson Syndrome (SJS)/Toxic Epidermal Necrolysis (TEN)

Raschi et al. [65] reported a case of rare and serious SJS/TEN, also known as Lyell syndrome, after the use of SGLT2 inhibitors. The findings suggest the isolated occurrence of SJS/TEN after commencing an SGLT2 inhibitor and imply that prompt recognition and stopping the offending medication are vital. SJS/TEN is a life-threatening condition of skin and mucous membrane lesions. SJS/TEN is characterized by a positive Nikolsky sign and the presence of tender mucous erosions affecting at least two different anatomic locations. The exact mechanism behind SGLT2-inhibitor-induced SJS/TEN is yet to be determined. Healthcare providers should monitor patients for signs of characteristic blisters and skin peeling. SJS/TEN is a rare CADR that has high mortality.

### 3.10. Other CADRs

The US FAERS Public Dashboard reported 4388 cases of skin and subcutaneous tissue adverse reactions after using SGLT2 inhibitors, with diabetic foot, skin rash, and skin ulcers accounting for 811, 765, and 617 cases, respectively [69]. According to post-marketing surveillance of SGLT2 inhibitors, infections, pruritus, photosensitivity, and urticaria topped the reported list of 1136 cases of skin and subcutaneous tissue adverse drug reactions [65]. Mellander et al. [67] reported hypersensitivity-related cutaneous adverse events associated with the commencement of dapagliflozin and described non-serious rash, eczema, dermatitis, and urticaria as their common findings. A pooled analysis by Mellander et al. [67] studied potential hypersensitivity-related skin events associated with dapagliflozin in a database from 13 clinical trials involving 4456 patients with T2DM treated with dapagliflozin 5–10 mg/day for up to a duration of 2 years. In total, 2.4% of patients treated with dapagliflozin were reported with hypersensitivity events, with the most common CADRs being rash, pruritus, and urticaria, followed by respiratory events including dyspnea and wheezing, and gastrointestinal events such as diarrhea and nausea [67]. Similarly, Filippas-Ntekouan et al. [68] and Yabe et al. [56] found generalized rash, urticaria, erythema, drug eruption, and eczema as the most common CADRs. Ikehara et al. [62] reported a case of bullous pemphigoid during treatment with ipragliflozin. There was an increased risk of developing psoriasis in patients with diabetes and renal diseases after commencing SGLT2 inhibitors [63]. Ma et al. [63] reported the risk of psoriasis and Mounsey et al. [64] reported candidiasis-induced inflammatory vulvitis with psoriasiform features in patients after commencing SGLT2 inhibitors.

### 3.11. Significance

Our work suggests that, in view of so many CADRs after the initiation of SGLT2 inhibitors, it is important to continuously monitor for CADRs during the use of SGLT2 inhibitors. It was observed that most CADRs generally appear within the first 2 weeks of commencing SGLT2 inhibitors [56,68], making it important to strictly monitor the patients for the first 15 days. Healthcare providers should carefully evaluate the benefits and risks of SGLT2 inhibitors and identify the population at a higher risk of developing CADRs. Patients have to be made aware from day 1 of commencing SGLT2 inhibitors to recognize early signs of any CADR including Fournier’s gangrene, fixed drug eruptions, SJS/TEN, pruritus, rashes, and other lesions. Regulatory agencies should closely monitor all reported adverse events as well as come up with strategies for the early detection and prevention of CADRs. More research is needed to study the mechanisms and the pathophysiology behind all the SGLT2-inhibitor-induced cutaneous reactions and findings.

Frequent monitoring and surveillance of adverse drug reactions should be performed to explore the association between various types of CADRs and the use of SGLT2 inhibitors. In the presence of appropriate pH, temperature, moisture, glucose concentration, and altered immunity and inflammatory responses in diabetics, bacteria can readily proliferate [92,93]. Patients with DM and peripheral vascular disease are at an increased risk of skin and soft-tissue infections. Dryden et al. [92] described the pathophysiology and burden of infection in this population. DM and peripheral vascular disease increase the risk of developing skin and soft-tissue infections by hampering the body’s natural defenses against infection [94]. These patients have diabetic neuropathy, a reduction in blood flow, and prolonged states of hyperglycemia in uncontrolled cases with high HbA1c. Nerve damage reduces sensation to trauma and infection. Reduced blood flow leads to reduced oxygen delivery and hampers wound healing and adequate immune responses. Sustained hyperglycemic states impair neutrophil function [94], increasing susceptibility to infection as well as the formation of biofilms by bacteria that make infections more resistant to treatment [92]. These patients generally present with foot ulcers, cellulitis, and abscesses in variable stages of severity [95], with common sites of serious infection being head and neck, biliary tract, urinary tract, skin, soft tissue, and bony structures of the feet [96]. This is important because skin and soft-tissue infections in patients with DM and peripheral vascular disease are associated with prolonged hospital stays, prolonged antibiotic treatment, amputations, financial burden, chronic pain, limited mobility, psychosocial issues, and high mortality [92,96]. If oral hypoglycemic agents are contributing to CADRs, then they can worsen the already-present skin and soft-tissue infections and further complicate the patients’ morbidity. Further studies are needed to identify any role of this group of drugs on any receptors similar to SGLT2 present in the skin and soft tissues, creating a glucose-rich environment that, in turn, would promote bacterial growth [97]. Studies have shown that, when prescribed in combination with metformin, the adverse effects were reported a little less, and this is partly attributed to the antimicrobial properties of metformin [98,99,100,101]. On the other hand, however, the combination of SGLT2 inhibitors and metformin has posed an increased risk of developing metabolic acidosis and diarrhea [102,103]. From the literature-based evidence of CADRs, the authors conclude that patients on SGLT2 inhibitors should be closely monitored for any form of newly developing skin lesions and should be consulted by a dermatologist.

## 4. Strengths and Limitations

One of the strengths of this review is the identification of a substantial number of CADRs reported with the commencement of SGLT2 inhibitors. A high number of Fournier’s gangrene cases is reported in patients who have used the SGLT2 inhibitor group of drugs with variable duration. Moreover, findings of fixed drug eruptions, SJS/TEN, drug-induced pruritus, and Sweet syndrome/acute febrile neutrophilic dermatosis have been linked with the commencement of SGLT2 inhibitors and subsided after discontinuing them. One limitation of this review is that, since any cutaneous adverse event is predominantly a self-reported one, our study cannot identify cases that are missed by self-reporting, especially the ones that are less troublesome to the patients. Under-reporting is the most important limitation of self-reported adverse drug reactions [104,105].

## 5. Conclusions

Apart from the cutaneous pathological processes inherent to T2DM and local reactions, contact dermatitis, and lipohypertrophy at insulin injection sites, there are a variety of CADRs in T2DM patients, and our work focused on CADRs linked to SGLT2 inhibitors, the latest and highly promising oral hypoglycemic agents with relatively fewer side effects and broader areas of benefits in the range of cardiovascular and renal systems. Most CADRs have unclear underlying pathophysiology mechanisms and they raise new questions upon existing knowledge such as the reasons behind the high predisposition for Fournier’s gangrene and pruritus after SGLT2 inhibitor use. In order to reduce complications, minimize hospitalizations, and improve patient care in the vulnerable population of DM patients, healthcare providers have to raise their awareness regarding CADRs to SGLT2 inhibitors. Healthcare providers should weigh the benefits vs. the risks of SGLT2 inhibitors, identify populations at higher risks of developing CADRs, and closely monitor the patients for the first 2 weeks of commencing SGLT2 inhibitors.

## Figures and Tables

**Table 1:** Key features of various SGLT2 inhibitor drugs.

Drug; BRAND	Available Doses; Initial Dose	Renal Impairment (eGFR is in ml/min/1.73 m^2^)	Hepatic Impairment	Drug-Specific Adverse Effect	Other Remarks
Canagliflozin; INVOKANA	100 mg, 300 mg; 100 mg OD	eGFR 45–59: do not exceed 100 mg/day eGFR < 45: do not initiate, discontinue	No adjustment in mild-to-moderate impairment; not recommended in severe impairment	Slight increase in the risk of bone fracture and lower limb amputation in those treated with canagliflozin Rarely severe hypersensitivity Rarely hyperkalemia Rarely pruritus	Digoxin co-administration increases digoxin exposure
Dapagliflozin; FORXIGA	5 mg, 10 mg; 5 mg OD	eGFR < 60: do not initiate, discontinue contraindicated in ESRD, dialysis	No adjustment in mild, moderate, or severe impairment	Slight increase in the risk of bladder cancer in those treated with dapagliflozin Rarely pruritus Rarely sweet syndrome	Mefenamic acid co-administration increases dapagliflozin exposure
Empagliflozin; JARDIANCE	10 mg, 25 mg; 10 mg OD	eGFR < 45: do not initiate, discontinue	No adjustment in mild, moderate, or severe impairment	Rarely severe hypersensitivity Rarely pruritus	-
Ertugliflozin; STEGLATRO	5 mg, 15 mg; 5 mg OD	eGFR < 60: do not initiate, discontinue contraindicated in ESRD, dialysis	No adjustment in mild-to-moderate impairment; not studied and not recommended in severe impairment	Slight increase in the risk of lower-limb amputation in those treated with ertugliflozin	-
Bexagliflozin; BEXA	20 mg; 20 mg OD	eGFR < 30: do not initiate, discontinue	No adjustment in mild-to-moderate impairment; not studied and not recommended in severe impairment	Slight increase in the risk of intravascular volume depletion in those treated with bexagliflozin	-
Ipraglflozin; SUGLAT	50 mg, 100 mg; 50 mg OD	eGFR < 30: do not initiate, discontinue	No adjustment in mild impairment; not studied and not recommended in moderate-to-severe impairment	Rarely bullous pemphigoid	-
Sotagliflozin; INPEFA	200 mg, 400 mg; 200 mg OD	eGFR < 30: do not initiate, discontinue	No adjustment in mild impairment; not studied and not recommended in moderate-to-severe impairment	Rarely diarrhea and gastrointestinal disturbances	-

**Table 2:** Summary of articles.

Publication	Drug	CADR	Time Since Drug Initiation	Patients Details	Other Remarks
Chi WC and Lim-Tio S, 2016 [37]	Dapagliflozin	Fournier’s gangrene	3 weeks	Male, 67 yrs	Case report
Kumar et al., 2017 [38]	Empagliflozin	Fournier’s gangrene	14 months	Male, 41 yrs	Case report
Omer et al., 2018 [39]	Dapagliflozin	Fournier’s gangrene	5 months	Male, 60 yrs	Case report
Onder et al., 2019 [40]	Dapagliflozin	Fournier’s gangrene	6 months	Male, 64 yrs	Case report
Nagano et al., 2019 [41]	Empagliflozin	Fournier’s gangrene	142 days	Male, 34 yrs	Case report
Rodler et al., 2019 [42]	Dapagliflozin	Fournier’s gangrene	4 years	Male, 39 yrs	Case report
Elshimy et al., 2019 [43]	Empagliflozin	Fournier’s gangrene	10 days	Male, 57 yrs	Case report
Elbeddini et al., 2020 [44]	Canagliflozin	Fournier’s gangrene	6 years	Male, 72 yrs	Case report
Ellegard L and Prytz M, 2020 [45]	Dapagliflozin	Fournier’s gangrene	1.5 years	Female, 52 yrs	Case report
Garcia-Garcia et al., 2020 [46]	Dapagliflozin	Fournier’s gangrene	<3 years	Male, 68 yrs	Case report
Kasbawala et al., 2020 [47]	Canagliflozin	Fournier’s gangrene	1 month	Female, 37 yrs	Case report
Moon et al., 2021 [48]	Dapagliflozin	Fournier’s gangrene	Unspecified	Male, 66 yrs	Case report
Suciu et al., 2024 [49]	Unspecified	Fournier’s gangrene	6 months	Male, 65 yrs	Case report
Hu et al., 2020 [50]	Multiple	Fournier’s gangrene	-	Multiple	Pharmacovigilance
Wang et al., 2020 [51]	Multiple	Fournier’s gangrene	-	Multiple	Nested case-control study
Tran et al., 2022 [52]	Multiple	Fournier’s gangrene	-	Multiple	FAERS database
Keskin et al., 2019 [53]	Dapagliflozin	fixed drug eruptions	2 weeks	Female, elderly	Case report
Damiani et al., 2016 [54]	Canagliflozin	fixed drug eruptions	2 weeks	Female, 64 yrs	Case report
Saito-Sasaki et al., 2017 [55]	Ipragliflozin	fixed drug eruptions	Unspecified	Female, 67 yrs	Case report
Yabe et al., 2015 [56]	Ipragliflozin	Rashes	Unspecified	Unspecified	Case report
Sakaeda et al., 2018 [57]	Ipragliflozin	Rashes	Unspecified	Unspecified	Case report
Vasapollo et al., 2018 [58]	Canagliflozin	Pruritus	Unspecified	Female, 61 yrs	Case report
Yau et al., 2022 [59]	Empagliflozin	Pruritus	2 weeks	Female, 65 yrs	Case report
Ali et al., 2021 [60]	Dapagliflozin	Pruritus	<4 weeks	Female, 47 yrs	Case report
Mattis et al., 2019 [61]	Dapagliflozin	Sweet syndrome	2 weeks	Female, 67 yrs	Case report
Ikehara et al., 2018 [62]	Ipragliflozin	Bullous pemphigoid	Unspecified	Unspecified	Case report
Ma et al., 2022 [63]	Multiple	Psoriasis	Unspecified	Unspecified	Case report
Mounsey et al., 2023 [64]	Multiple	Psoriasiform vulvitis	Unspecified	Unspecified	Case report
Raschi et al., 2017 [65]	Multiple	Multiple	-	Multiple	FAERS database
Fadini et al., 2019 [16]	Multiple	Multiple	-	Multiple	Pharmacovigilance
Bersoff-Matcha et al., 2019 [66]	Multiple	Multiple	-	Multiple	FAERS database
Mellander et al., 2016 [67]	Dapagliflozin	Multiple	-	Multiple	Pooled analysis
Filippas-Ntekouan et al., 2018 [68]	Multiple	Multiple	-	Multiple	Pooled analysis
FDA 2021 [69]	Multiple	Multiple	-	Multiple	FAERS database
